# Action and lumbar forces during hospital bed handling: A comparative study

**DOI:** 10.1177/10519815261429454

**Published:** 2026-03-26

**Authors:** Claus Backhaus, Chris Schröer, Lorenz Müller, Niels Hinricher

**Affiliations:** 1 Center for Ergonomics and Medical Engineering, FH Münster University of Applied Sciences, Steinfurt, Germany; 2 German Social Accident Insurance for Health and Welfare Services, Berlin, Germany

**Keywords:** musculoskeletal disorders, pushing, pulling, healthcare professionals, spine, movement analysis, posture

## Abstract

**Background:**

Nursing staff show a higher-than-average prevalence of musculoskeletal disorders compared with other occupational groups. Moving hospital beds is among the most physically demanding nursing tasks. However, the influence of hospital bed design on the musculoskeletal strain experienced by nursing personnel during bed movement has not been sufficiently investigated.

**Objective:**

This study examined the action and compressive forces (L5/S1) generated while moving twelve different hospital beds.

**Methods:**

Eight participants moved the beds along a test course resembling a hospital ward. Action forces were measured using three-dimensional force measurement grips, and a motion analysis system recorded the participants’ postures during the task. Based on these recordings, compressive forces at the lumbosacral junction (L5/S1) were calculated using the biodynamic model “The Dortmunder”.

**Results:**

Median peak action forces ranged from 157.3 N to 240.0 N during bed acceleration and from 49.6 N to 92.4 N during steady movement. In several cases, the calculated compressive forces at L5/S1 exceeded the guideline value of 1.8 kN for 60-year-old female employees, indicating a moderately to substantially increased load for nursing staff.

**Conclusion:**

Design features such as a fifth caster on the hospital bed can reduce physical strain. Bed movers or active casters should be employed for frequent bed transport, long-distance pushing, or when the staff includes older employees or individuals with preexisting musculoskeletal conditions.

## Introduction

Nurses are among the occupational groups most frequently affected by musculoskeletal disorders (MSDs).^1^ In addition to patient mobilization, moving heavy medical equipment can also place substantial strain on the lower back. In particular, transporting patients in hospital beds imposes considerable loads on the lumbar spine.^2^ However, studies quantifying these loads and systematically comparing different hospital bed designs remain scarce.

Previous research on hospital bed transport by nursing staff has primarily focused on evaluating powered assistance systems^3–5^ and assessing the effectiveness of specific design features.^6,7^ Nevertheless, empirical evidence quantifying the mechanical or physiological strain experienced by nursing professionals during bed transport remains limited.

Leban et al. (2020) analyzed hand forces during the movement of a loaded bed and reported the highest values during the initial phase of movement. However, their study was restricted to longitudinal and transverse force components, and participants were not nursing staff.^8^ Brütting et al. (2017) examined action and compressive forces (L5/S1) while moving hospital beds; however, their study was limited to a single bed model, nonrepresentative flooring, and a small, non-nursing sample.^2^

Design modifications such as a fifth wheel or steering lock have consistently been shown to reduce transport effort and physical strain while improving handling.^7,9,10^ Similarly, powered drive systems have been demonstrated to lower hand forces, lumbar compression, and perceived exertion.^11,12^ Studies investigating the high prevalence of MSDs among nurses have identified risk factors such as the frequent movement of heavy loads and years of professional experience.^13–15^ Given the limited quantification of lumbar loads associated with these disorders, more detailed analyses of spinal stress during hospital bed transport are warranted.

The present study therefore records and compares action and lumbar compressive forces while pushing and pulling twelve different hospital beds under realistic working conditions.

## Methods

### Experimental design

The selection of hospital beds was based on a previously conducted market analysis. Twelve beds (B1–B12) were included in the study. Ten of these beds were equipped with a fifth caster, which enhanced maneuverability and directional stability. Two beds (B1 and B7) did not include a fifth caster (Table 1).

**Table 1.** List of hospital beds tested (B1–B12).

Alias	Manufacturer	Model	Fifth caster	Caster type
B1	Wissner-Bosserhoff	Eleganza 1	–	150 mm twin wheel
B2	Malsch	Impulse	✓	150 mm twin wheel
B3	Stiegelmeyer	Seta Pro	✓	150 mm twin wheel
B4	Stiegelmeyer	Evario	✓	150 mm twin wheel
B5	Stiegelmeyer	Puro	✓	150 mm twin wheel
B6	Völker	S966	✓	150 mm twin wheel
B7	Völker	S962-2	–	150 mm single wheel
B8	Wissner-Bosserhoff	Image 3	✓	150 mm twin wheel
B9	Wissner-Bosserhoff	Eleganza 2	✓	150 mm twin wheel
B10	Arjo	Enterprise 8000X	✓	150 mm twin wheel
B11	Stryker	SV-2	✓	150 mm twin wheel
B12	Hillrom	900	✓	150 mm twin wheel

Eight caregivers (six males and two females) participated in the study. Their mean age was 27 ± 2 years, with an average body weight of 78 ± 11 kg and a mean height of 179 ± 9 cm. All participants had prior experience in moving hospital beds, wore their usual professional footwear, and provided written informed consent. None reported any musculoskeletal complaints at the time of the study.

The sequence of hospital beds was determined using a computer-generated simple randomization procedure, and each bed was subsequently moved three times by each caregiver along a predefined test course within the hospital ward. To minimize potential fatigue and learning effects, a minimum rest period of 15 min was maintained between repeated trials for each participant.

The test course comprised two sections: (1) maneuvering the bed out of a patient room while pulling backward, and (2) pushing the bed for 12 m along the hospital corridor, including a 90° turn after 6 m. During all tests, the beds were loaded with 107 kg to simulate a heavy patient corresponding to the 90th percentile of male body weight. The hospital ward floor was covered with standard linoleum flooring, as commonly used in clinical environments.

### Action forces

**Figure 1.**
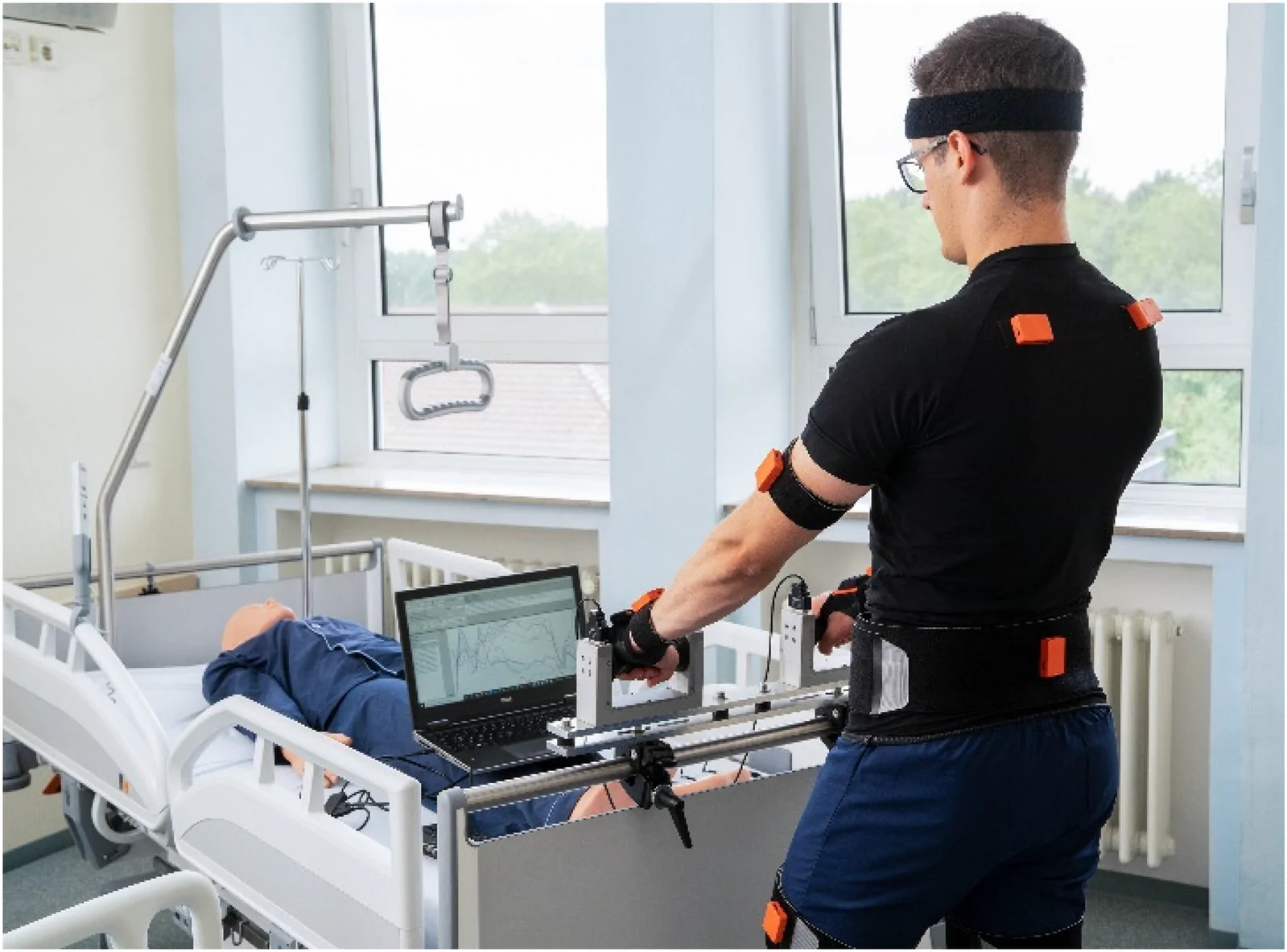
Hospital bed equipped with three-dimensional force transducers and caregiver wearing the motion analysis system while maneuvering a bed out of a patient room.

Three-dimensional force transducers (Model 9809A, Kistler, Germany) were used to measure the action forces (Figure 1). Each transducer was attached to the hospital bed and individually adjusted to the caregivers’ height so that the grip height was centered between the wrist and the elbow, with the arms extended downward.^2^

The initial and constant forces were derived from the measured action forces.^16^ In this study, the initial forces corresponded to the maximum forces required to initiate bed movement, whereas the mean forces represented the constant forces necessary to sustain motion. The measured values were compared with the limit values specified in IEC 60601-2-52 (160 N for initial forces and 85 N for constant forces).^17^

### Compressive forces L5/S1

To determine the compressive forces, the caregivers’ postures were recorded using a wearable motion analysis system (Xsens Awinda, Movella Inc., USA) (Figure 1). The compressive forces at the lumbosacral junction (L5/S1) were calculated from the action forces and body postures using the biodynamic model “The Dortmunder”.^18^ As with the action forces, both the maximum and mean compressive forces were determined for each hospital bed.

The data were processed using the WIDAAN software (Version 8/21) developed by the Institute for Occupational Safety and Health of the German Social Accident Insurance in Berlin. The revised Dortmund guideline value of 1.8 kN for 60-year-old female employees was used to evaluate the L5/S1 compressive forces.^19^ This reference value also served to identify potential overloads in older workers.

### Statistical analysis

Maximum and mean values were calculated in two steps. First, the three trials of each participant were averaged. Second, these individual means were averaged across all participants for each bed. This procedure was applied to both the measured action forces and the estimated compressive forces at L5/S1.

For all outcome variables (mean and maximum values of action and compressive forces), repeated-measures analyses of variance were conducted with the within-subject factor *bed* (12 levels: B1–B12). Mauchly's test of sphericity was used to verify the assumption of sphericity, and Greenhouse-Geisser corrections were applied where necessary. When significant main effects were observed (p < .05), Sidak-adjusted pairwise comparisons were performed. To account for the small sample size and potential violations of normality, nonparametric Friedman tests were also conducted. When these tests revealed significant effects, Bonferroni-adjusted Wilcoxon signed-rank tests were applied for pairwise comparisons.

All statistical analyses were performed using IBM SPSS Statistics (Version 29; IBM Corp., Armonk, NY, USA).

## Results

Detailed descriptive statistics (mean ± SD and 95% CI) and complete test results for all hospital beds are provided in Supplementary Tables S1 (maneuvering out of a patient room) and S2 (pushing along the hospital corridor).

### Action forces

#### Maneuvering the bed out of a patient room

**Figure 2.**
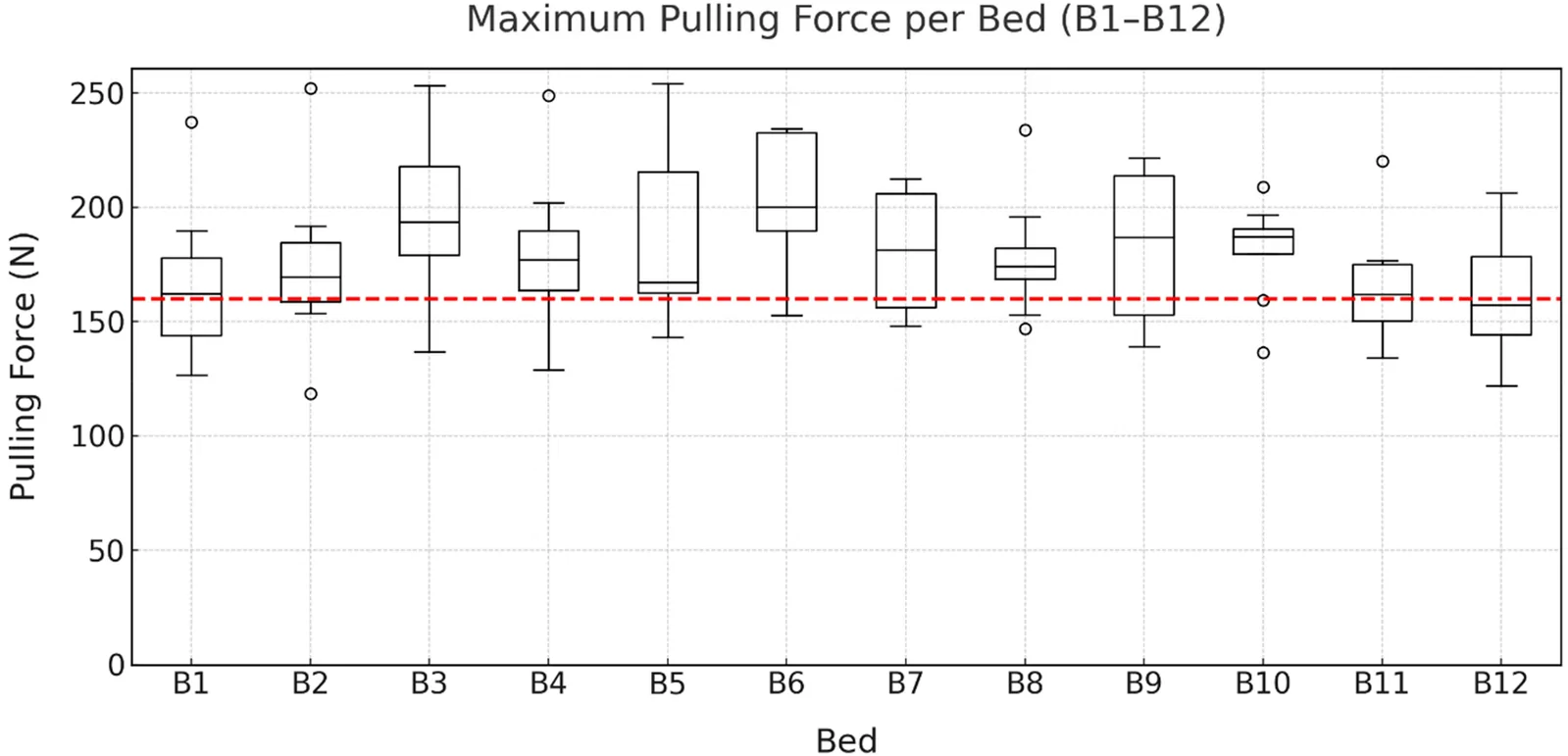
Maximum pulling forces when maneuvering the beds (B1–B12) by eight participants. The IEC 60601-2-52 limit of 160 N is indicated in red.

Figure 2 illustrates the distribution of maximum pulling forces required to maneuver each bed (B1–B12) out of a patient room. The median maximum pulling forces ranged from 157.3 N (144.2–178.3 N; B12) to 200.2 N (189.9–232.5 N; B6) across the different beds. The medians for beds B1, B11, and B12 were close to or below the IEC 60601-2-52 limit of 160 N, whereas for all other beds, this limit was at least partly and, in some cases, clearly exceeded.

The repeated-measures ANOVA (Mauchly's test: W < .001, p < .001; Greenhouse-Geisser correction: ε = .377) revealed no statistically significant effect of *bed* on maximum pulling force (F(4.14, 28.99) = 2.48, p = .064, ηp^2^ = .261, n = 8). However, the supplementary Friedman test indicated significant overall differences between the beds (χ^2^(11) = 24.65, p = .010). Bonferroni-adjusted pairwise comparisons did not identify any statistically significant differences (all p_a_d_j_ > .05).

**Figure 3.**
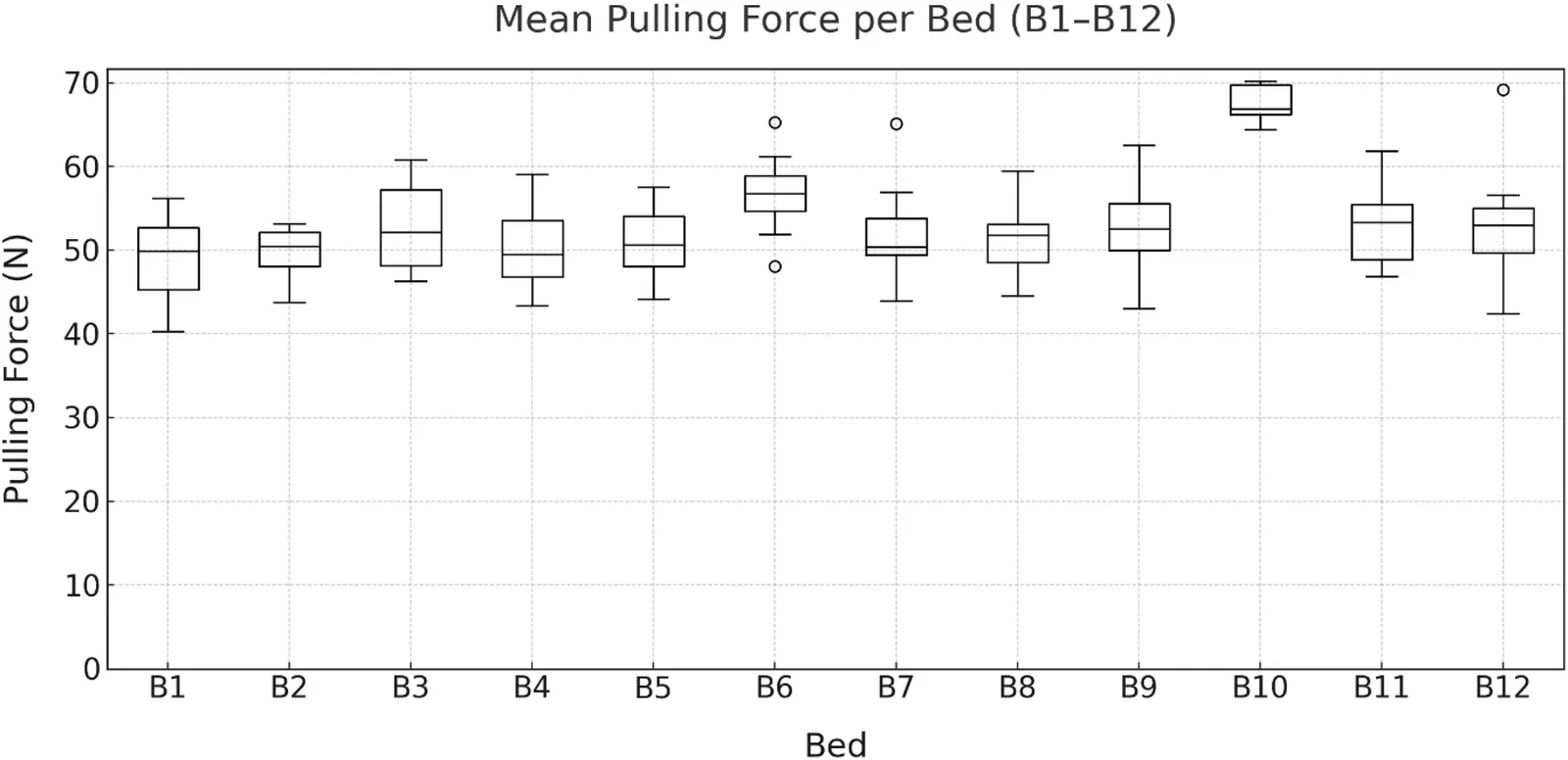
Mean pulling forces when maneuvering the beds (B1–B12) by eight participants.

Figure 3 illustrates the distribution of mean pulling forces required to maneuver each bed (B1–B12) out of a patient room. The median of the mean pulling forces ranged from 49.6 N (46.8–53.6 N; B4) to 66.9 N (66.3–69.8 N; B10) across the different beds. None of the beds exceeded the IEC 60601-2-52 limit of 85 N.

Mean pulling force differed significantly by bed (repeated-measures ANOVA, Greenhouse-Geisser corrected: F(3.94, 27.57) = 13.13, p < .001, ηp^2^ = .652). Post-hoc comparisons showed that bed B10 required significantly higher forces than beds B1–B5 and B7–B11, whereas the differences compared to beds B6 and B12 were not significant.

#### Pushing the bed 12 m along the hospital corridor

**Figure 4.**
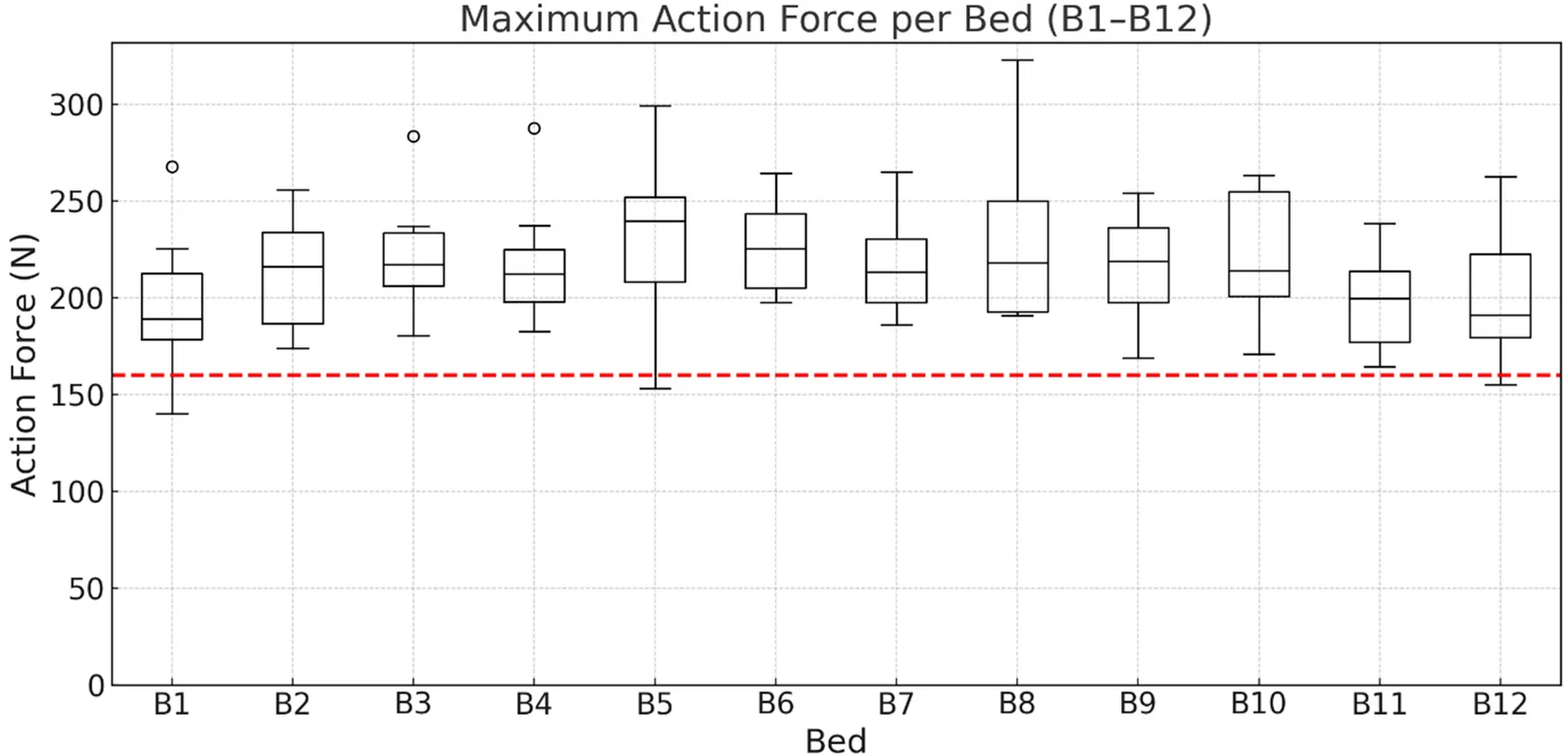
Maximum pushing forces when moving the beds (B1–B12) along the hospital corridor by eight participants. The IEC 60601-2-52 limit of 160 N is indicated in red.

When pushing the beds along the hospital corridor, the maximum forces ranged from 140 N (B1) to 322.9 N (B8). The median maximum forces varied between 189.1 N (178.6–212.9 N; B1) and 240.0 N (208.1–252.1 N; B5). As shown in Figure 4, all median values exceeded the IEC 60601-2-52 limit of 160 N.

No statistically significant bed effect was found for maximum pushing force (repeated-measures ANOVA, Greenhouse-Geisser corrected: F(2.91, 20.34) = 1.97, p = .153, ηp^2^ = .219). Although a Friedman test suggested an overall difference (χ^2^(11) = 19.75, p = .049), Bonferroni-adjusted post hoc comparisons did not reveal significant pairwise differences.

**Figure 5.**
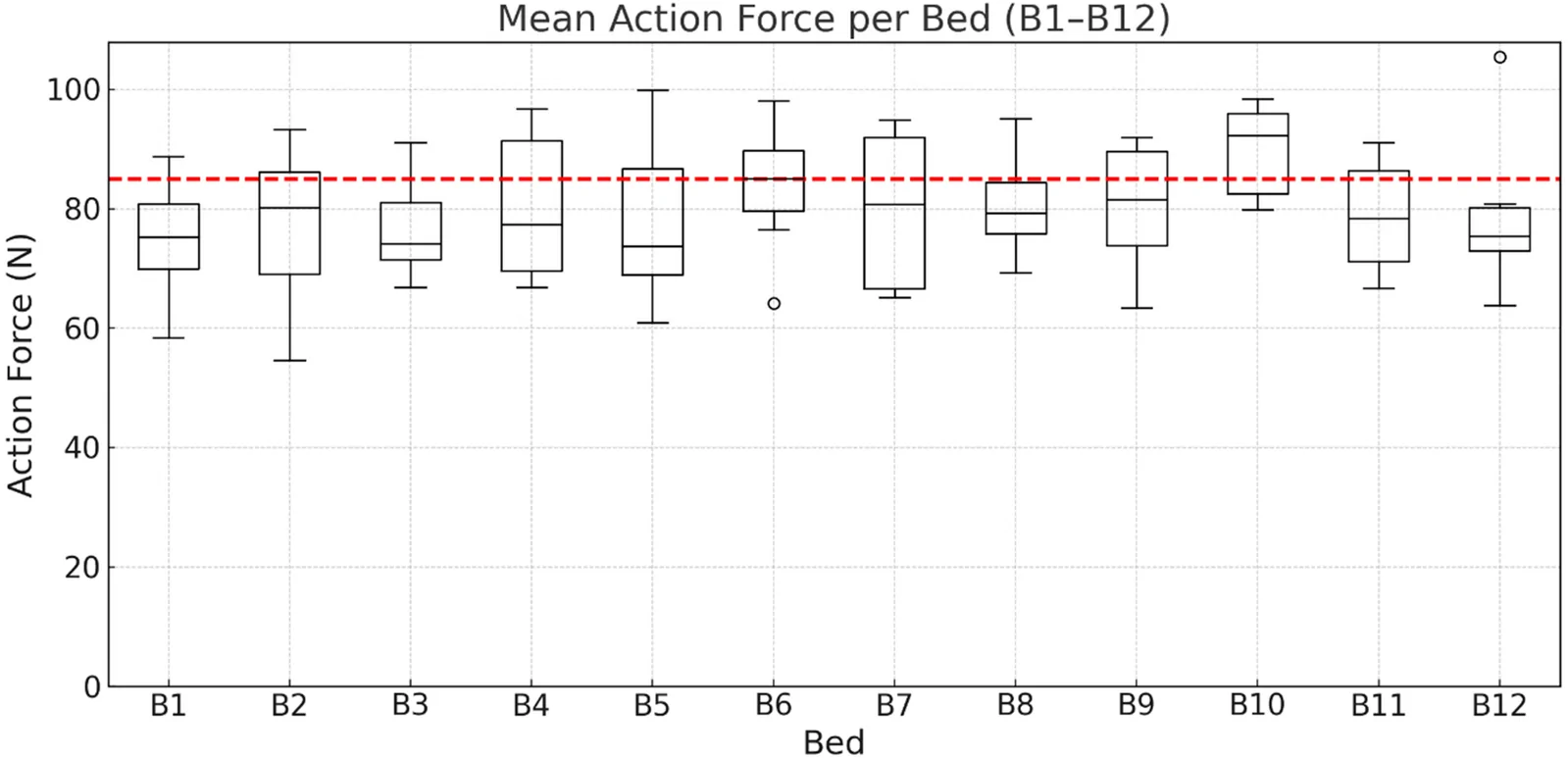
Mean pushing forces when moving the beds (B1–B12) along the hospital corridor by eight participants. The IEC 60601-2-52 limit of 85 N is indicated in red.

As shown in Figure 5, the mean pushing forces when moving the beds along the hospital corridor ranged from 54.6 N (B2) to 99.9 N (B5). The median for B10 exceeded the IEC 60601-2-52 limit of 85 N, with a value of 92.4 N (82.5–96.0 N), whereas B6 was essentially at the limit with 85.1 N (79.6–89.8 N). The medians for all other beds remained below the specified limit.

Mean pushing force differed significantly by bed (repeated-measures ANOVA, Greenhouse-Geisser corrected: F(3.60, 25.37) = 3.12, p = .036, ηp^2^ = .308). Post hoc comparisons indicated higher forces for bed B10 than for bed B1, and no other pairwise differences were statistically significant.

### Compressive forces L5/S1

#### Maneuvering the bed out of the patient room

**Figure 6.**
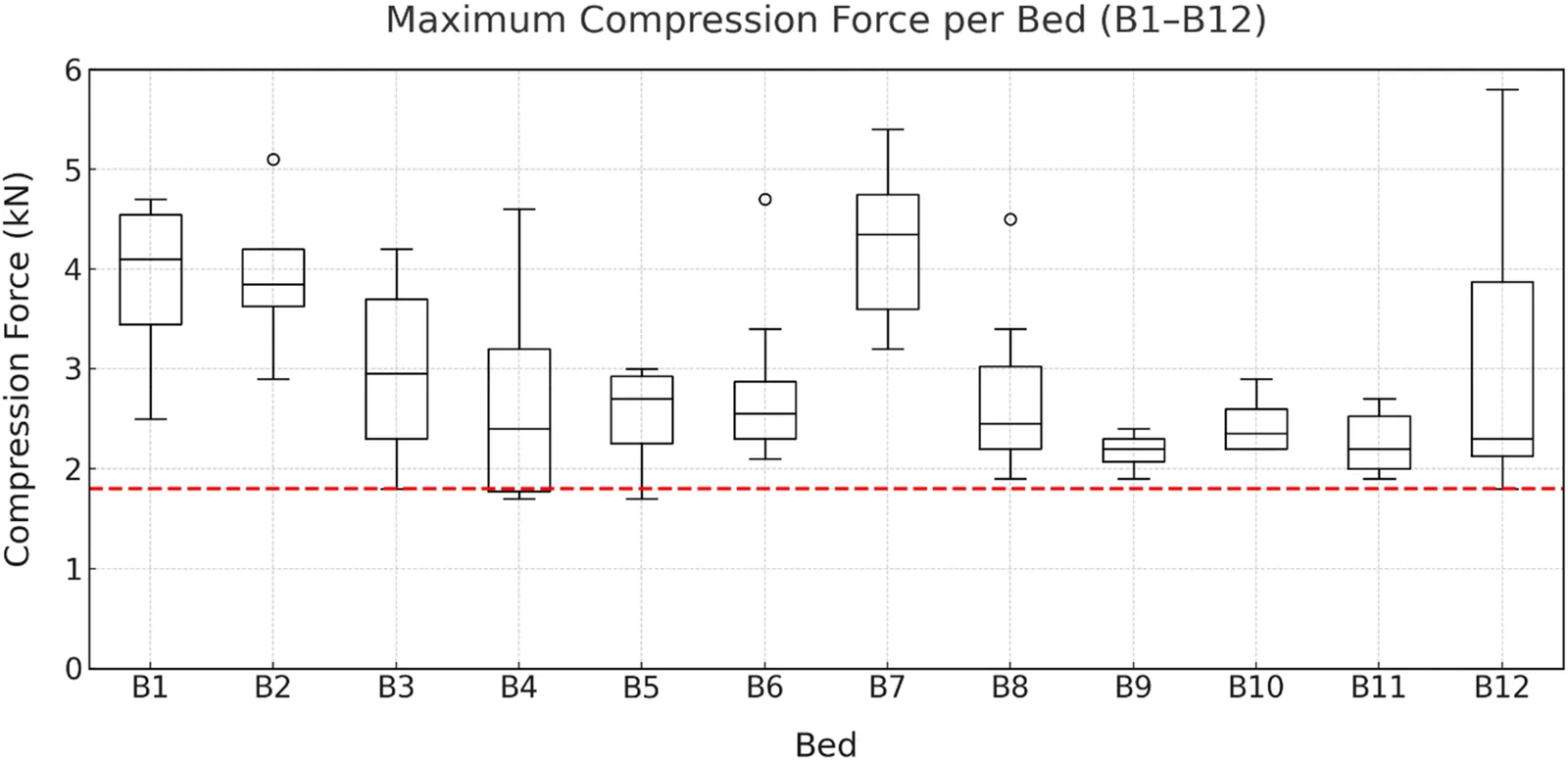
Maximum compressive forces when maneuvering the beds (B1–B12) by eight participants. The revised Dortmund reference value for 60-year-old female employees (1.8 kN) is indicated in red.

As shown in Figure 6, the maximum compressive forces when maneuvering the beds out of a patient room ranged from 1.7 kN (B4 and B5) to 5.8 kN (B12). All median values exceeded the reference limit of 1.8 kN. Particularly high median values were observed for beds B1, B2, and B7, with 4.1 kN, 3.9 kN, and 4.4 kN, respectively.

Maximum L5/S1 compressive force differed significantly by bed (repeated-measures ANOVA, Greenhouse-Geisser corrected: F(3.73, 26.12) = 7.02, p < .001, ηp^2^ = .501). Post-hoc comparisons showed higher values for bed B7 than for beds B4, B5, and B9–B11; beds B1 and B2 also showed higher values than beds B9–B11.

**Figure 7.**
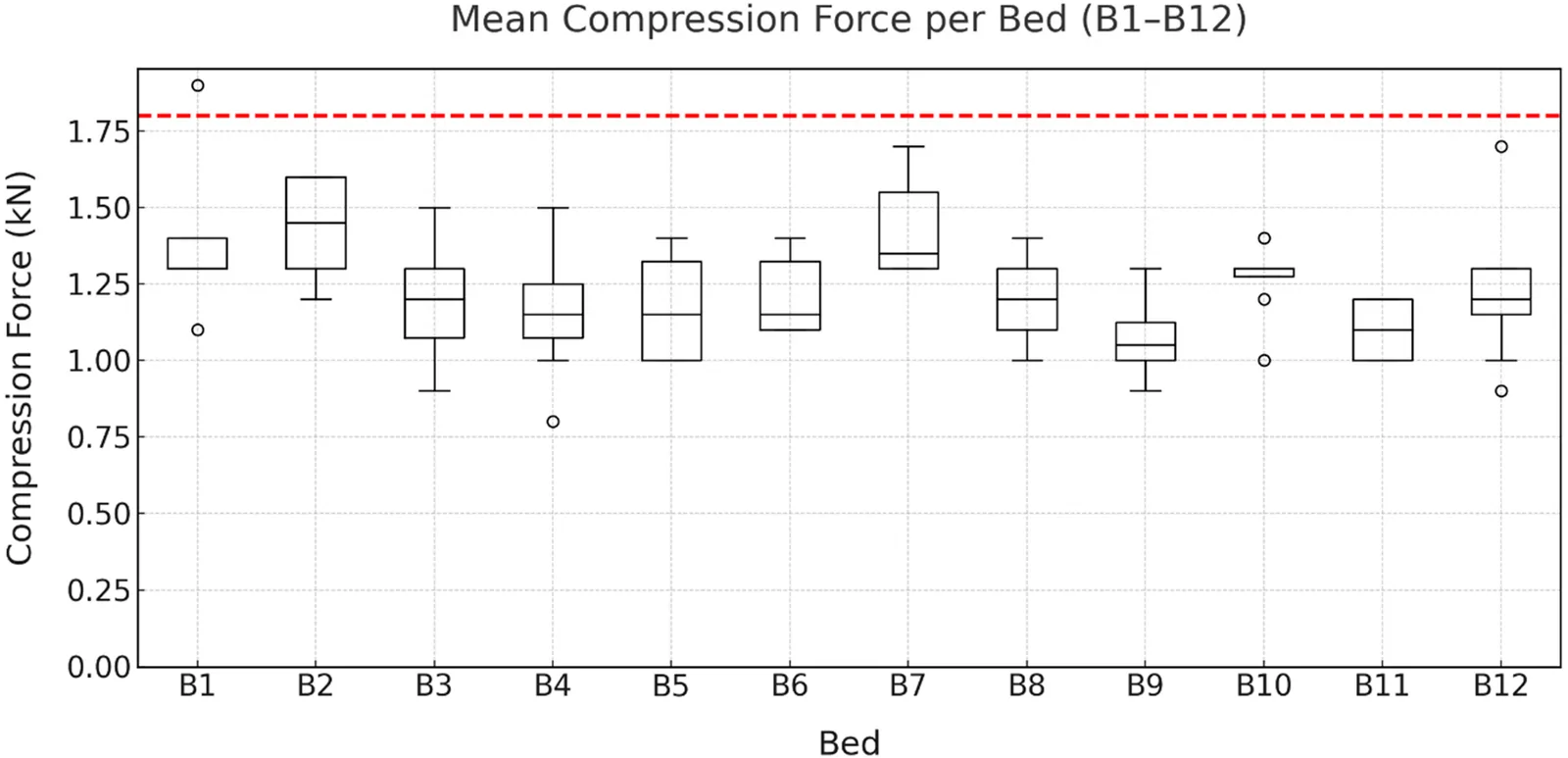
Mean compressive forces when maneuvering the beds (B1–B12) by eight participants. The revised Dortmund reference value for 60-year-old female employees (1.8 kN) is indicated in red.

Figure 7 illustrates the mean compressive forces when maneuvering the beds out of a patient room. Median values ranged between 1.10 kN and 1.45 kN. Accordingly, the reference limit for mean compressive forces (1.8 kN) was not exceeded by any of the tested beds.

Mean L5/S1 compressive force differed significantly by bed (repeated-measures ANOVA, Greenhouse-Geisser corrected: F(3.64, 25.45) = 4.09, p = .013, ηp^2^ = .369). Post hoc comparisons identified a single significant difference, with bed B2 showing higher values than bed B11.

#### Pushing the bed 12 m along the hospital corridor

**Figure 8.**
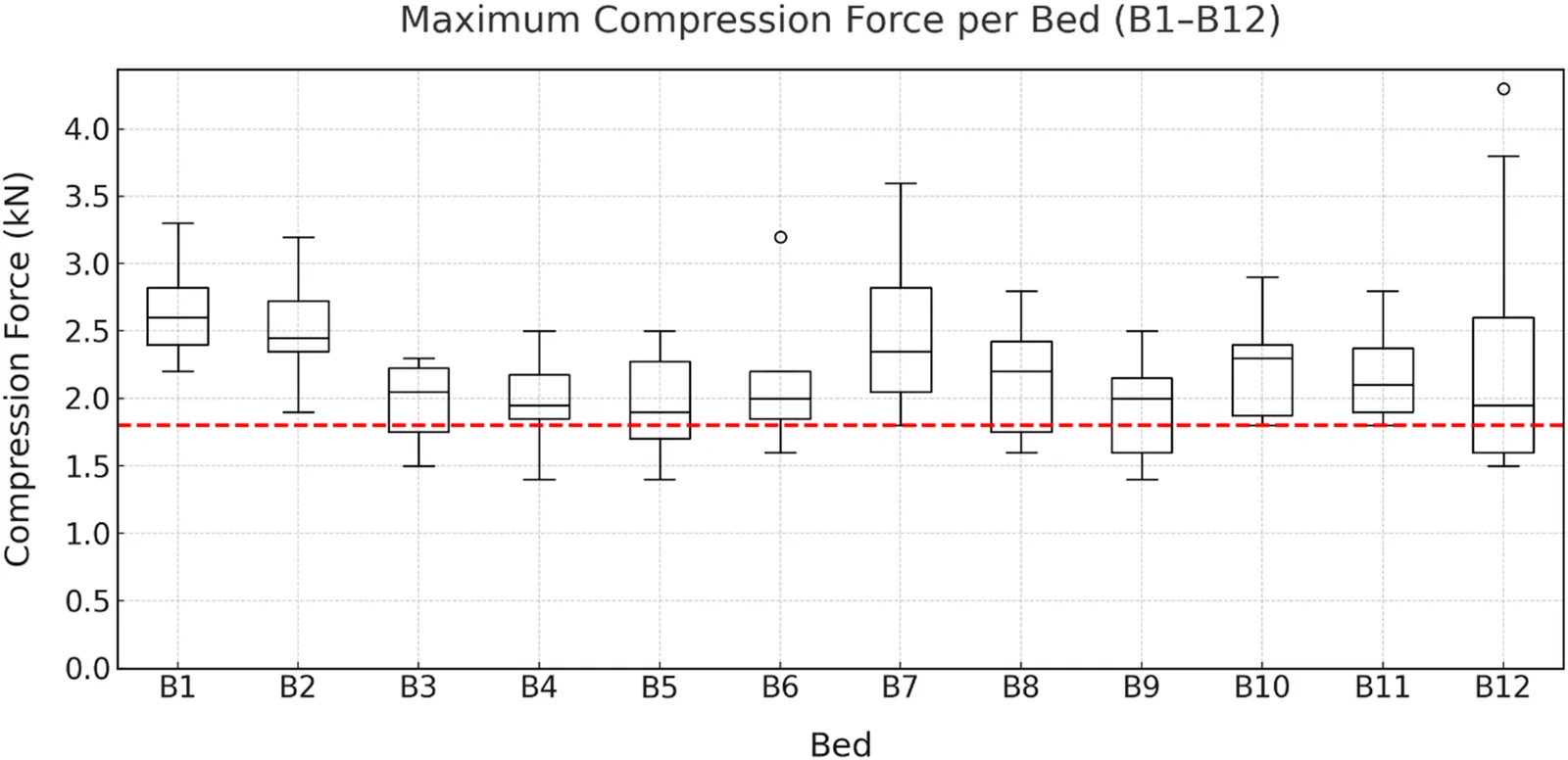
Maximum compressive forces when pushing the beds along the hospital corridor (B1–B12) by eight participants. The revised Dortmund reference value for 60-year-old female employees (1.8 kN) is indicated in red.

The results presented in Figure 8 show the maximum compressive forces when pushing the beds along the hospital corridor. These values ranged from 1.4 kN (B4, B5, and B9) to 4.3 kN (B12). The median values varied between 1.9 kN (1.7–2.3 kN; B5) and 2.6 kN (2.4–2.8 kN; B1), thereby exceeding the reference limit for all beds.

A repeated-measures ANOVA (Greenhouse-Geisser corrected) showed no statistically significant bed effect on maximum L5/S1 compressive force (F(2.40, 16.90) = 2.54, p = .101, ηp^2^ = .308). A Friedman test indicated an overall difference across beds (χ^2^(11) = 25.72, p = .007), but Bonferroni-adjusted pairwise comparisons were not significant (all p_adj > .05).

**Figure 9.**
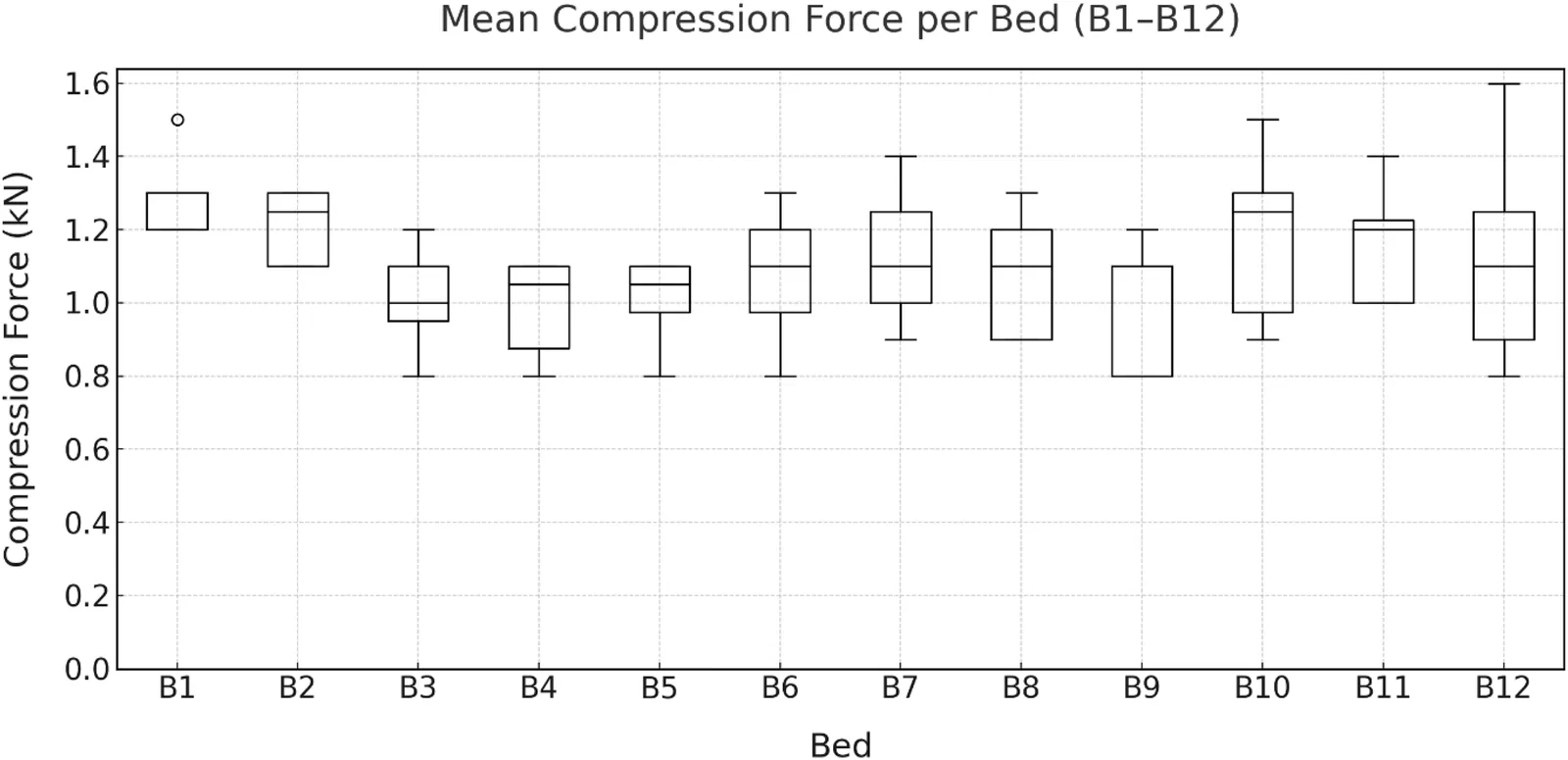
Mean compressive forces when pushing the beds along the hospital corridor (B1–B12) by eight participants.

Figure 9 illustrates the mean compressive forces when pushing the beds along the hospital corridor. The recorded values ranged from 0.8 kN to 1.6 kN. The medians of the participants’ mean compressive forces varied between 1.0 kN (B3) and 1.3 kN (B1, B2, and B10), and the reference limit of 1.8 kN was not exceeded for any of the beds.

Mean L5/S1 compressive force differed significantly by bed (repeated-measures ANOVA, Greenhouse-Geisser corrected: F(2.59, 18.12) = 5.89, p = .007, ηp^2^ = .457). Post-hoc comparisons indicated that bed B2 produced significantly higher values than beds B3–B5, and bed B11 produced higher values than beds B4 and B9.

## Discussion

The results indicated that while maneuvering the beds out of a patient room and pushing them along the hospital corridor, high maximum action forces were recorded, clearly exceeding the limit of 160 N specified by IEC 60601-2-52. Only the mean forces observed when maneuvering the beds out of the patient room remained below the 85 N threshold, which applies to the forces required to sustain movement.

Complementary guidance for manual pushing/pulling tasks is provided by ISO 11228-2 (including Amendment 1:2022), which places these force requirements within a broader ergonomic risk assessment context (e.g., force magnitude, frequency, duration, handle height, and environmental conditions).^20^ Because patient transports in clinical practice varies substantially (e.g., longer travel distances, different frequencies, and building-related factors such as door thresholds, ramps, uneven flooring, and cornering), ISO 11228-2 yields task-specific acceptable forces rather than a single threshold. Under realistic assumptions for hospital bed transport, the acceptable force levels may span a broad range (approximately 40–110 N for sustained pushing and 110–210 N for initial pushing, depending on the parameter combination). A comparison of these ranges with our measurements shows that the ISO-based guideline values, especially for peak demands, are exceeded in many situations.

Overall, the participants were exposed to greater action forces when pushing the beds along the hospital corridor than when pulling them backward out of the patient room. Because of the narrow doorway width, the bed inside the patient room had to be positioned with relatively high precision, resulting in slower movement and consequently lower initial forces. In contrast, when pushing the bed along the corridor, such spatial restrictions were absent, allowing participants to accelerate the beds more freely, thereby increasing the initial forces.

The measured action forces were lower than those reported in the study by Brütting et al.^2^ According to the authors, this difference can be attributed to variations in the test procedure. For example, in the study by Brütting et al., a used bed without a fifth castor was pushed along the corridor of an office building by untrained participants. The maneuvering was not performed from a patient room but was simulated by moving the bed several times through 90° in an open area without spatial constraints.

The study by Leban et al.^7^ reported lower mean action forces when pushing a bed along the corridor of a hospital ward. However, a major methodological difference from the present study is that the action forces were measured only in two planes (x- and y-axes), preventing the calculation of a resultant force vector.

The calculated maximum compressive forces at L5/S1 demonstrated that the revised Dortmund guideline value of 1.8 kN was reached or exceeded for all twelve beds, both during maneuvering and when pushing along the hospital corridor. In contrast, the mean compressive forces in both scenarios remained clearly below this threshold. This finding is interpreted as indicating a slightly increased to increased physical load. The L5/S1 data further show that spinal strain was greater when maneuvering the beds inside the patient room than when pushing them along the hospital corridor. During maneuvering, caregivers must apply greater lateral action forces, leading to lateral flexion and torsion of the trunk. Despite the lower overall action forces compared with pushing, these lateral components result in higher compressive loads at L5/S1.

Beds B1, B2, and B7 produced particularly high compressive forces at L5/S1. Beds B1 and B7 were not equipped with a fifth castor. In all other beds, the fifth castor was centrally located beneath the chassis at the bed's center of gravity. The fifth castor is locked in position before the bed is moved, which facilitates turning and enables straight-ahead movement. In the case of bed B2, however, the fifth castor was installed by the manufacturer outside the bed's center of gravity, causing it to drag on the floor when cornering. This misalignment results in increased action forces and consequently higher compressive loads at L5/S1.

In this study, the hospital beds were moved by a single person rather than by two caregivers, as recommended in occupational safety guidelines.^21^ When hospital beds are moved by two people, it is typically done to complete the task more quickly or with greater precision, as is often the case when moving a bed out of a patient room. However, more time-consuming activities—such as transferring a patient to another ward or transporting them for examination—are commonly performed by one caregiver due to staff shortages and economic considerations. An exception occurs when medical requirements necessitate the involvement of multiple caregivers, for instance when transferring intensive care patients together with several pieces of medical equipment. It can be expected that moving beds with two caregivers would reduce both action forces and compressive loads at L5/S1, although not necessarily by half, due to potential coordination inefficiencies known as the Ringelmann effect. The extent of this load reduction and the influence of a fifth castor during two-person bed transport should be examined in future studies.

Electrically operated bed transport systems (bed movers) represent an effective option for reducing physical load, particularly during long-distance transport. In such systems, the hospital bed is coupled to an electric trolley controlled by the caregiver. However, bed movers also present certain drawbacks, including their high purchase cost, limited compatibility with beds from different manufacturers, and increased structural dimensions, which can complicate transportation in confined spaces such as elevators. A promising alternative involves active or power-assisted bed castors equipped with motion sensors that detect bed movement. Once motion is detected, a motor integrated into the castor hub assists propulsion.^11^

The main limitation of the present study is its small sample size. Nevertheless, significant differences between the beds were identified, particularly in the compressive forces between models with and without a fifth castor. Another limitation is the gender imbalance among participants: in contrast to the typical gender distribution in hospital settings, a greater number of men than women participated. Leban et al. (2019) reported that women exerted significantly higher mean pushing forces than men (75 N vs. 62 N).^22^ However, it can be assumed that men, owing to their higher body mass and their ability to apply more body weight against the bed during the initiation phase, may generate higher peak forces. Future studies should examine gender-related differences in both pushing and pulling hospital beds under controlled experimental conditions.

The study was conducted at a single site, which may limit the generalizability of its findings, as environmental and organizational factors—such as flooring materials, ward layouts, and local work practices—may differ across healthcare settings.

The standardized patient weight of 107 kg used in this study represented a typically heavy patient. In clinical practice, however, the total load—particularly for intensive care unit beds—can be considerably higher due to the added weight of attached medical equipment. Future research should therefore assess spinal loading when hospital beds are subjected to their maximum permissible total weight.

## Conclusion

Moving hospital beds imposes considerable physical strain, particularly on the lumbar spine. During bed maneuvering out of patient rooms, the threshold for spinal compressive forces was exceeded for all twelve beds tested. However, the findings also indicate that incorporating a fifth castor substantially reduces these compressive loads. Therefore, newly manufactured hospital beds should be equipped with a fifth castor to promote a safer and more ergonomically sustainable working environment. Additional preventive measures are recommended to further reduce mechanical stress, especially during frequent or long-distance bed transports, movements on inclines, and tasks performed by older employees or those with pre-existing musculoskeletal conditions. Such measures include the preferential use of technical aids, such as electrically operated bed movers or power-assisted bed castors.

## Supplemental Material

sj-docx-1-wor-10.1177_10519815261429454 - Supplemental material for Action and lumbar forces during hospital bed handling: A comparative study Supplemental material, sj-docx-1-wor-10.1177_10519815261429454 for Action and lumbar forces during hospital bed handling: A comparative study by Claus Backhaus, Chris Schröer, Lorenz Müller and Niels Hinricher in WORK
