# Supplementary material for: Action and lumbar forces during hospital bed handling: A comparative study
Source: Work. 2026 Mar 26;85(1):52–61. doi: 10.1177/10519815261429454 (PMC13550738; doi:10.1177/10519815261429454)
Supplement: Supplementary file 1 [file sj-docx-1-wor-10.1177_10519815261429454.docx]

**Supplementary Materials**

**Table S1.** Comparison of mean and maximum action forces and spinal compressive forces when maneuvering the hospital beds out of the patient room.

| **Measure** | **Bed** | **Mean** | **SD** | **Median** | **95% CI lower** | **95% CI upper** | **sig. difference** |
| --- | --- | --- | --- | --- | --- | --- | --- |
| **Max pulling force (N)** | B1 | 166.4 | 35.7 | 162.2 | 141.7 | 191.2 | — |
|  | B2 | 174.7 | 38.3 | 169.5 | 148.2 | 201.2 | — |
|  | B3 | 196.8 | 35.3 | 193.6 | 172.3 | 221.2 | — |
|  | B4 | 180.4 | 35.1 | 176.9 | 156.0 | 204.7 | — |
|  | B5 | 185.9 | 38.8 | 167.1 | 159.0 | 212.8 | — |
|  | B6 | 203.1 | 29.2 | 200.2 | 182.8 | 223.3 | — |
|  | B7 | 181.1 | 28.2 | 181.3 | 161.6 | 200.7 | — |
|  | B8 | 178.6 | 26.9 | 174.1 | 159.9 | 197.2 | — |
|  | B9 | 183.9 | 33.4 | 186.8 | 160.8 | 207.1 | — |
|  | B10 | 181.3 | 22.8 | 187.2 | 165.6 | 197.1 | — |
|  | B11 | 165.7 | 26.6 | 161.9 | 147.3 | 184.1 | — |
|  | B12 | 161.4 | 27.3 | 157.3 | 142.5 | 180.3 | — |
| **Mean pulling force (N)** | B1 | 49.1 | 5.6 | 49.9 | 45.1 | 53.0 | < B10 |
|  | B2 | 49.5 | 3.6 | 50.5 | 47.0 | 52.0 | < B10 |
|  | B3 | 52.8 | 5.5 | 52.2 | 49.0 | 56.6 | < B10 |
|  | B4 | 50.4 | 5.5 | 49.6 | 46.6 | 54.2 | < B10 |
|  | B5 | 50.8 | 4.9 | 50.6 | 47.4 | 54.2 | < B10 |
|  | B6 | 56.7 | 5.3 | 56.8 | 53.1 | 60.4 | — |
|  | B7 | 52.3 | 6.3 | 50.4 | 47.9 | 56.7 | < B10 |
|  | B8 | 51.4 | 4.4 | 51.8 | 48.3 | 54.4 | < B10 |
|  | B9 | 52.5 | 6.0 | 52.6 | 48.3 | 56.6 | < B10 |
|  | B10 | 67.5 | 2.2 | 66.9 | 66.0 | 69.0 | >B1-B5 and B7-B11 |
|  | B11 | 53.0 | 5.1 | 53.3 | 49.5 | 56.6 | < B10 |
|  | B12 | 53.2 | 8.1 | 53.1 | 47.6 | 58.7 | — |
| **Max compressive force (kN)** | B1 | 3.9 | 0.8 | 4.1 | 3.3 | 4.5 | >B9-B11 |
|  | B2 | 3.9 | 0.7 | 3.9 | 3.4 | 4.4 | >B9-B11 |
|  | B3 | 3.0 | 0.9 | 3.0 | 2.4 | 3.6 | — |
|  | B4 | 2.7 | 1.1 | 2.4 | 1.9 | 3.4 | <B7 |
|  | B5 | 2.5 | 0.5 | 2.7 | 2.2 | 2.9 | <B7 |
|  | B6 | 2.8 | 0.9 | 2.6 | 2.2 | 3.4 | —- |
|  | B7 | 4.3 | 0.8 | 4.4 | 3.7 | 4.8 | >B4, B5, B9-B11 |
|  | B8 | 2.7 | 0.9 | 2.5 | 2.1 | 3.3 | — |
|  | B9 | 2.2 | 0.2 | 2.2 | 2.1 | 2.3 | < B7 |
|  | B10 | 2.4 | 0.3 | 2.4 | 2.2 | 2.6 | < B7 |
|  | B11 | 2.3 | 0.3 | 2.2 | 2.0 | 2.5 | < B7 |
|  | B12 | 3.1 | 1.5 | 2.3 | 2.1 | 4.1 | — |
| **Mean compressive force (kN)** | B1 | 1.4 | 0.2 | 1.3 | 1.2 | 1.5 | — |
|  | B2 | 1.4 | 0.2 | 1.5 | 1.3 | 1.5 | > B11 |
|  | B3 | 1.2 | 0.2 | 1.2 | 1.1 | 1.3 | — |
|  | B4 | 1.2 | 0.2 | 1.2 | 1.0 | 1.3 | — |
|  | B5 | 1.2 | 0.2 | 1.2 | 1.1 | 1.3 | — |
|  | B6 | 1.2 | 0.1 | 1.2 | 1.1 | 1.3 | — |
|  | B7 | 1.4 | 0.2 | 1.4 | 1.3 | 1.6 | — |
|  | B8 | 1.2 | 0.1 | 1.2 | 1.1 | 1.3 | — |
|  | B9 | 1.1 | 0.1 | 1.1 | 1.0 | 1.2 | — |
|  | B10 | 1.3 | 0.1 | 1.3 | 1.2 | 1.3 | — |
|  | B11 | 1.1 | 0.1 | 1.1 | 1.0 | 1.2 | < B11 |
|  | B12 | 1.2 | 0.2 | 1.2 | 1.1 | 1.4 | — |

**Table S2.** Comparison of mean and maximum action forces and spinal compressive forces when pushing the hospital beds along the corridor.

| **Measure** | **Bed** | **Mean** | **SD** | **Median** | **95 % CI lower** | **95 % CI upper** | **sig. difference** |
| --- | --- | --- | --- | --- | --- | --- | --- |
| **Max pulling force (N)** | B1 | 196.2 | 38.6 | 189.1 | 169.5 | 223.0 | — |
|  | B2 | 213.7 | 30.8 | 216.1 | 192.3 | 235.1 | — |
|  | B3 | 222.1 | 30.9 | 217.1 | 200.7 | 243.5 | — |
|  | B4 | 218.0 | 33.4 | 212.6 | 194.8 | 241.1 | — |
|  | B5 | 229.4 | 46.5 | 240.0 | 197.2 | 261.6 | — |
|  | B6 | 226.4 | 24.5 | 225.4 | 209.3 | 243.4 | — |
|  | B7 | 216.9 | 25.9 | 213.3 | 199.0 | 234.8 | — |
|  | B8 | 230.1 | 45.5 | 218.4 | 198.6 | 261.7 | — |
|  | B9 | 216.2 | 30.4 | 219.0 | 195.2 | 237.3 | — |
|  | B10 | 222.0 | 34.2 | 214.0 | 198.4 | 245.7 | — |
|  | B11 | 197.5 | 25.7 | 199.6 | 179.7 | 215.3 | — |
|  | B12 | 202.5 | 36.7 | 191.2 | 177.1 | 228.0 | — |
| **Mean pulling force (N)** | B1 | 75.1 | 10.0 | 75.3 | 68.1 | 82.0 | < B10 |
|  | B2 | 76.7 | 13.5 | 80.2 | 67.4 | 86.1 | — |
|  | B3 | 76.9 | 9.0 | 74.2 | 70.7 | 83.1 | — |
|  | B4 | 79.9 | 12.0 | 77.4 | 71.6 | 88.3 | — |
|  | B5 | 77.6 | 13.2 | 73.8 | 68.4 | 86.7 | — |
|  | B6 | 83.6 | 10.2 | 85.1 | 76.5 | 90.7 | — |
|  | B7 | 80.0 | 12.8 | 80.8 | 71.2 | 88.9 | — |
|  | B8 | 80.8 | 8.2 | 79.3 | 75.2 | 86.5 | — |
|  | B9 | 80.6 | 10.7 | 81.6 | 73.2 | 88.0 | — |
|  | B10 | 90.1 | 7.5 | 92.4 | 84.9 | 95.3 | > B1 |
|  | B11 | 78.5 | 9.1 | 78.4 | 72.2 | 84.8 | — |
|  | B12 | 78.2 | 12.2 | 75.4 | 69.8 | 86.7 | — |
| **Max compressive force (kN)** | B1 | 2.7 | 0.4 | 2.6 | 2.4 | 2.9 | — |
|  | B2 | 2.5 | 0.4 | 2.5 | 2.2 | 2.8 | — |
|  | B3 | 2.0 | 0.3 | 2.1 | 1.8 | 2.2 | — |
|  | B4 | 2.0 | 0.4 | 2.0 | 1.7 | 2.2 | — |
|  | B5 | 2.0 | 0.4 | 1.9 | 1.7 | 2.3 | — |
|  | B6 | 2.1 | 0.5 | 2.0 | 1.8 | 2.4 | — |
|  | B7 | 2.5 | 0.7 | 2.4 | 2.0 | 3.0 | — |
|  | B8 | 2.1 | 0.4 | 2.2 | 1.8 | 2.4 | — |
|  | B9 | 1.9 | 0.4 | 2.0 | 1.7 | 2.2 | — |
|  | B10 | 2.2 | 0.4 | 2.3 | 2.0 | 2.5 | — |
|  | B11 | 2.2 | 0.4 | 2.1 | 1.9 | 2.4 | — |
|  | B12 | 2.4 | 1.1 | 2.0 | 1.6 | 3.1 | — |
| **Mean compressive force (kN)** | B1 | 1.3 | 0.1 | 1.3 | 1.2 | 1.4 | — |
|  | B2 | 1.2 | 0.1 | 1.3 | 1.1 | 1.3 | > B3-B5 |
|  | B3 | 1.0 | 0.1 | 1.0 | 0.9 | 1.1 | < B2 |
|  | B4 | 1.0 | 0.1 | 1.1 | 0.9 | 1.1 | < B2, B11 |
|  | B5 | 1.0 | 0.1 | 1.1 | 0.9 | 1.1 | < B2 |
|  | B6 | 1.1 | 0.2 | 1.1 | 1.0 | 1.2 | — |
|  | B7 | 1.1 | 0.2 | 1.1 | 1.0 | 1.3 | — |
|  | B8 | 1.1 | 0.2 | 1.1 | 1.0 | 1.2 | — |
|  | B9 | 1.0 | 0.2 | 1.1 | 0.9 | 1.1 | < B11 |
|  | B10 | 1.2 | 0.2 | 1.3 | 1.0 | 1.3 | — |
|  | B11 | 1.2 | 0.2 | 1.2 | 1.1 | 1.3 | > B4, B9 |
|  | B12 | 1.1 | 0.3 | 1.1 | 0.9 | 1.3 | — |
